# Effect of bone graft granule volume on postoperative fusion after lumber spinal internal fixation: A retrospective analysis of 82 cases

**DOI:** 10.12669/pjms.345.14971

**Published:** 2018

**Authors:** Jianxue Hao, Chongchao Yan, Suoli Liu, Pengfa Tu

**Affiliations:** 1 Jianxue Hao, Baoding First Hospital, Baoding 071000, P. R. China; 2 Chongchao Yan, Baoding First Hospital, Baoding 071000, P. R. China; 3 Suoli Liu, Baoding First Hospital, Baoding 071000, P. R. China; 4 Pengfa Tu, Baoding First Hospital, Baoding 071000, P. R. China

**Keywords:** Intervertebral bone graft, Internal fixation, Spinal fusion

## Abstract

**Objective:**

To investigate the effect of bone graft volume on postoperative fusion and symptom improvement in lumbar posterior lumbar fusion and internal fixation.

**Methods:**

A total of 82 patients receiving pedicle screw rod system internal fixation with Cage bone graft fusion in the First Hospital of Baoding City, Hebei Province were selected and randomly divided into three groups. The excised autologous laminar bones were bitten into different sizes of bone fragments. And different sizes of bone grafts were implanted during the operation. Group-A (n=28) was implanted by bone graft granule with the average volume of 0.2 cm^3^, Group-B (n=27) was implanted by bone graft granule with the average volume of 0.1 cm^3^, and Group-C (n=27) was implanted by bone graft granule with the average volume of 0.05 cm^3^. The bone graft granule volume, clinical effect, bone graft fusion rate and intervertebral space height were compared.

**Results:**

The three groups had significantly different bone graft granule volumes (P<0.05), but similar intervertebral bone graft total volumes and Cage heights (P>0.05). In the final follow-up, VAS and ODI of low back pain and two lower limbs pain significantly reduced compared with those before surgery (P<0.05), but the three groups had similar results (P>0.05). The bone graft fusions of Group-B one and two years after surgery were significantly higher than those of Group-A and Group-C, and the values of Group-A exceeded those of Group-C (P<0.05). In the final follow-up, the intervertebral space height change of Group-B was significantly smaller than those of Group-A and Group-C (P<0.05).

**Conclusion:**

Size of bone graft granule has no significant effect on postoperative symptoms. However, middle-sized volume bone graft granule (0.1 cm^3^/granule) showed increased postoperative intervertebral fusion rate and reduced intervertebral space height loss in our study.

## INTRODUCTION

Lumbar vertebrae is a key hub for human trunk activity, and almost all physical activity increases the burden on the lumbar spine. Nowadays, the society has entered the era of high-speed information tehcnology. With the rapid changes in people’s lifestyle, the prevalence of degenerative diseases of lumbar vertebrae has increased significantly. The incidence of degenerative diseases of the lumbar spine is mainly middle-aged and old, and it has a trend of younger age, which seriously affects Patient’s work and quality of life.

Lumbar posterior lumbar interbody fusion is a common method for the treatment of a variety of lumbar degenerative diseases, and has achieved good clinical results. Spinal pedicle screw rod system internal fixation with bone graft fusion and posterior interbody cage (abbreviated as Cage therafter) with rigid fixation in the preliminary stage has been widely used to treat lumbar intervertebral disc protrusion, spinal stenosis, and other lumbar degenerative diseases.^1^ The purpose is to restore the normal alignment of the lumbar spine, relieve the symptoms of lumbar nerve root compression and rebuild the stability of the spine. It has been reported in the literature that posterior lumbar interbody fusion (PLIF) has obvious advantages in biomechanics and clinical practice.^2^,^3^ Cloward^4^ first reported the use of posterior interbody fusion in the laminectomy. Although there are many implantable bone materials available for interbody fusion, there are different opinions on the choice of bone graft volume after clinical placement of Cage.^5^,^6^ Indigenous research is still rare on such research topics. In this study, patients with pedicle screw system and Cage bone grafting were divided according to the intraoperative interbody bone graft volume, and their clinical effects were compared, and evaluate the effects of bone graft granule volume on postoperative fusion.

## METHODS

This study has been approved by the ethics committee of our hospital, and written consent has been obtained from all patients. A total of 82 patients receiving pedicle screw rod system internal fixation with Cage bone graft fusion in the First Hospital of Baoding City, Hebei Province were randomly divided into three groups, and implanted with bone graft granules of different sizes during surgery. Group-A (n=28) was implanted with bone graft granule with the average volume of 0.20 cm^3^, Group-B (n=27) was implanted with bone graft granule with the average volume of 0.10 cm^3^, and Group-C (n=27) was implanted with bone graft granule with the average volume of 0.05 cm^3^. The three groups had similar gender ratio, age, course of disease, segmental lesions and other baseline clinical data (P>0.05). Indications included chronic, severe, disabling lumbar spine keyboard degenerative low back pain with or without radiculopathy; once less lumbar spondylolisthesis; reoperation after discectomy; lumbar spondylolysis without slippage; lumbar instability Caused by low back and leg pain; lumbar instability caused by lumbar and leg pain; ineffective after long-term conservative treatment.

### Surgical methods

All patients underwent general anesthesia, the patient was placed in a prone position, the sterile towel was routinely sterilized, and the surgical space was determined by C-arm fluoroscopy. We made an incision of about 5 cm via the posterior approach with surgical gap as the center, and incised the skin and subcutaneous tissue successively, retaining supraspinal and interspinous ligaments. Afterwards, we peeled off bilateral paravertebral muscles to expose bilateral vertebral plates and zygopophysis, conducted laminectomy and decompression for the affected side, removed ligamentum flavum exposed the endorhachis, nerve root, and pulled the dural sac and nerve root aside. After exposure of the intervertebral disc and posterior longitudinal ligament, we excised the prominent lumbar intervertebral disc tissue, explored the decompressed narrow lateral recess and nerve root canal, and fully relieved the compressed adhesive nerve root. Then we used slot cutter to expand the intervertebral space, and struck off the intervertebral disc and cartilage endplate until the bone end-plate. The excised autologous vertebral bones are cut into different sizes of bone fragments, and some are directly implanted into the intervertebral space with a bone graft, and the rest are used to fill a single interbody fusion cage of the corresponding model and obliquely tapped. Intervertebral space. It was followed by internal fixation with pedicle screws and rods. Hemostasis was achieved with bipolar coagulation and the wound was washed with normal saline. Wound was closed in layers over drain tubes. The drainage tubes were pulled out 24-48 hour after surgery, and lumbar X-ray examination was carried out on the next day to confirm the outcomes. The patients were encouraged to sit up with braces and to move around 3-4 days after surgery. After stitches were removed, the patients were discharged from the hospital two weeks after surgery, and regularly followed up in clinics.

### Observation indices

Bone grafting granule trimming and volume measurement during surgery were completed by one operator. The autopsy bone plate was trimmed using a rongeur, and the soft tissue attached to the bone surface was peeled off, and the autologous bone was trimmed into bone granules of different sizes according to different groups. Each bone granule was trimmed into the same size, put into a measuring cylinder with the volume of 10 ml, and added normal saline successively with one ml as unit until the bone granules were completely submerged. Then the reading of solution inside the measuring cylinder minus the volume of normal saline was the total volume of bone granules. The total volume divided by the number of bone granules was the average volume of each bone granule.

### Clinical assessment

Low back pain and two lower limbs pain were evaluated by using visual analogue score (VAS) before and after surgery, and function recovery was assessed by using Oswestry disability index (ODI).^7^

### Imaging assessment

At the time of follow-up, the lumbar spine was placed on the lateral radiograph of the lumbar spine, and the left and right lower facet joint images of the same vertebral body were superimposed on the lateral radiograph, to reduce errors caused by rotation. The intervertebral space heights in the front, middle and back of surgical section were measured and averaged, with the superior lumbar transverse diameter as reference to avoid error amplification.

### Statistical analysis

All data were analyzed with SPSS18.0 statistical software and expressed as mean ± standard deviation (x ± s). Comparisons among groups were performed using analysis of variance, and pairwise comparisons were conducted with the SNK test. The rank sum test or χ^2^ test was employed to compare numerical data. P<0.05 was considered statistically significant.

## RESULTS

The patient’s incision healed well after surgery, without early-stage complications. They were followed up for 24-48 months, 35 on average.

For Group-A, the average bone graft granule volume was (0.21±0.07) cm^3^, the total volume of intervertebral bone implantation was (6.7±1.2) cm^3^ and Cage height was (10.7± 1.0) mm. For Group-B, the average bone graft granule volume was (0.12±0.06) cm^3^, the total volume of intervertebral bone implantation was (6.9±1.8) cm^3^ and Cage height was (10.1± 0.9) mm. For Group-C, the average bone graft granule volume was (0.05±0.03) cm^3^, the total volume of intervertebral bone implantation was (6.8±1.4) cm^3^ and Cage average height was (10.4 ± 0.9) mm. The three groups had significantly different bone graft granule volumes (P<0.05), but similar total volumes of intervertebral bone implantation and Cage heights (P>0.05).

### Clinical assessment

The three groups had similar VAS and ODI of low back pain and two limbs pain before surgery (P>0.05). In the final follow-up, the indices of each group significantly reduced compared with those before surgery (P<0.05), without significant inter-group differences though (P>0.05) (Table-I).

**Table-I:** Low back pain VAS, two limbs pain VAS and ODI of each group before and after surgery (x ± s).

Group	Case number	Low back pain VAS		Two limbs pain VAS		ODI	

		Before surgery	Final follow-up	Before surgery	Final follow-up	Before surgery	Final follow-up
A	28	7.85±2.58	2.12±1.13	6.93±1.88	2.01±1.82	62.8±14.4	19.8±10.4
B	27	7.58±2.43	2.43±0.98	7.02±1.69	1.87±1.32	60.2±12.8	18.3±11.7
C	27	7.36±1.95	2.38±1.40	6.89±2.10	1.92±1.57	64.5±15.3	20.2±14.3

### Imaging assessment

**Fig.1:**
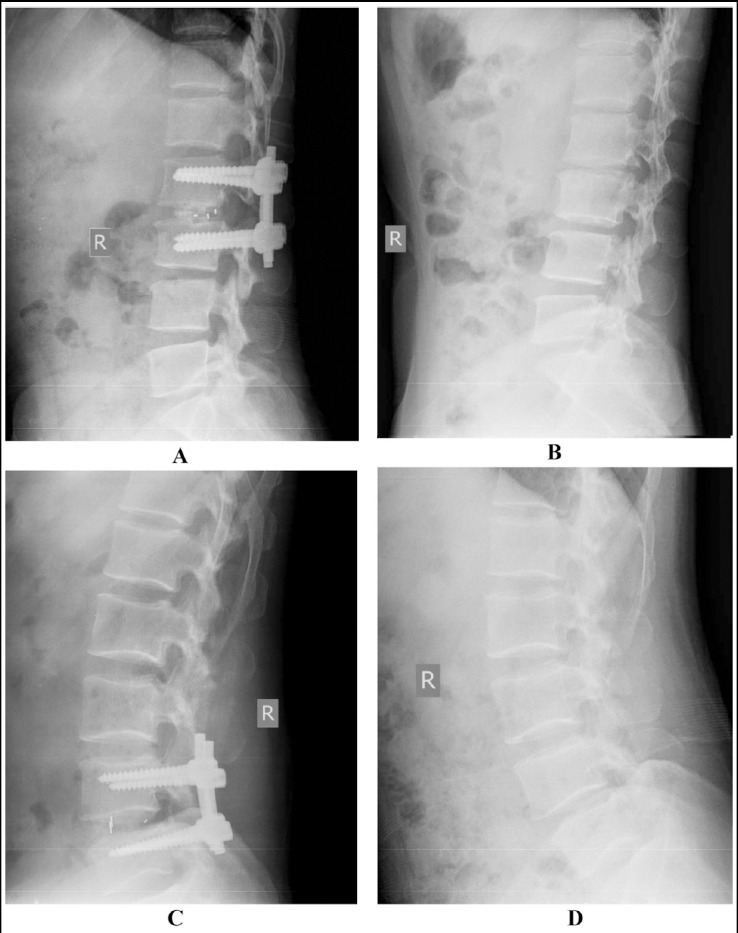
Imaging changes of typical cases before and two years after surgery. **A and C:** Pre-operative lateral X-ray film; **B and D:** Lateral X-ray film after bone graft fusion 2 years after operation.

Bone fusion rate = number of bone graft fusion cases / total number of cases in the group x 100%. X-ray film re-examination after one year of operation shows 22 patients with bone graft fusion in Group-A (78.6%); 24 patients with bone graft fusion in Group-B (88.9%); 18 patients with bone graft fusion in Group-C (66.7%); 23 patients with bone graft fusion in Group-A (82.1%) after two years of operation; 25 patients with bone graft fusion in Group-B (92.6%); 19 patients with bone graft fusion in Group-C (70.4%). Bone graft fusion rate in Group-B is obviously higher than Group-A and Group-C. The comparison differences between groups have statistical significance (P<0.05) (Fig.1).

The intervertebral space heights of each group after surgery and in the final follow-up significantly increased compared with those before surgery (P<0.05). Compared with immediate after surgery, the intervertebral space height changes of Group A-C in the final follow-up were (8.7±1.7) mm, (10.1±1.8) mm and (8.8±1.7) mm respectively. Group-B had significantly smaller changes than those of Group-A and Group-C (P<0.05). The differences between Group-A and Group-C were not statistically significant (P>0.05) (Table-II).

**Table-II:** Intervertebral space height of each group before and after surgery (x ± s)

Group	Case number	Before surgery /mm	Immediate after surgery /mm	Final follow-up/mm
A	28	7.7±1.8	11.1±1.7^1)^	8.7±1.7^1,3)^
B	27	7.4±1.9	10.7±1.4^1)^	10.1±1.8^1,2)^
C	27	7.8±2.1	11.4±1.5^1)^	8.8±1.7^1-3)^

1) Compared with pre-operation, P=0.018,

2) Compared with Group-A, P=0.022,

3) Compared with Group-B, P=0.028.

## DISCUSSION

Posterior spinal pedicle screw rod system internal fixation with bone graft fusion and Cage has been widely used to treat lumbar intervertebral disc protrusion, spinal stenosis, and other lumbar degenerative diseases. The purpose is to restore the normal alignment of the lumbar spine, relieve the symptoms of lumbar nerve root compression and rebuild the stability of the spine.^8^ In the final follow-up, all patients had fused without pseudarthrosis formation, internal fixation loosening or breakdown, or other complications. Good graft bed preparation, quality and quantity of bone graft blocks play important roles in intervertebral fusion.^9^,^10^ With the clinical application of polyether ether ketone (PEEK) and nano-materials such as polyether ether ketone (PEEK) with elastic modulus equivalent to vertebral bone and good compatibility with tissue, some scholars use locally excised vertebrae. Plate articular process, vertebral body posterior margin hyperplasia, bone smashing into bone graft to obtain autologous bone particles to avoid iliac bone grafting. In recent years, more and more studies have shown that the treatment of some degenerative lumbar diseases by PLIF can achieve good clinical results, while reducing complications and minimizing interference with spinal stability. At present, there is a lack of research on the bone grafting volume in the clinical research, and more scholars will focus on the attention of bone grafting. Limited bone grafting can affect intervertebral fusion. Allogeneic bone, xenogenic bone or bone morphogenetic protein (BMP) can be added to increase intervertebral fusion rate. However, factors such as disease transmission, inflammatory reaction and high cost make many users concerned about it. The size of the bone graft is more susceptible to the surgeon’s control, and this factor also plays an important role in the subsequent healing of the intervertebral. In this group of patients, we divided 82 patients into three groups of bone graft particles with different bone graft volume. In this study, the size of bone graft granule barely affected postoperative symptoms. Nevertheless, the postoperative fusion rate of middle-sized bone graft granule was significantly higher than those of smaller and larger bone graft bone granules. Regarding postoperative intervertebral height loss, middle-sized bone graft granule had obvious advantages.

Lumbar posterior pedicle screw fixation combined with Cage support bone graft fusion, such a cage can scan the X-ray, no artifacts during CT scan, can clearly and accurately observe the interbody fusion. The disadvantage is that it is brittle and easily causes damage to the cage, which in turn causes chemical reactions in the tissue. At present, most of the clinical use is the new material polymer polyetheretherketone (PEEK), which has outstanding advantages, can pass X-ray, does not affect CT and MRI examination, has good elastic modulus, and the stress shielding effect is more fusion than other materials. Small, not easily broken, corrosion-resistant and biocompatible, which greatly increases the intervertebral fusion rate. Most bone graft granules used in lumbar posterior pedicle screw fixation with Cage supporting bone graft fusion are cancellous bones which collapse easily when bearing strong strength.^11^ During bone graft prophase inflammatory reaction as well as anaphase callus reconstruction and remodeling, osteoblasts can absorb necrotic bone and bone tissue outside the stress axis while forming and connecting callus.^12^ After implantation of large bone granules, considerable gaps are produced during bone granule accumulation and mutual extrusion, causing bone trabecula breakage. The destruction of own grid structure promotes the destruction and resorption of implanted bone, being adverse to postoperative stability and intervertebral fusion. Ha KY et al.^13^ It has been found through CT scanning that bone graft volume in the intervertebral space decreased with elapsed time. Herein, the immediate postoperative intervertebral heights of patients had lost to various extents compared with those in the final follow-up, being consistent with the abovementioned descriptions.

Regardless, small-sized bone graft granules invisibly prolong the time for fiber joint formation between bone granules and end-plate due to severe bone lamella structural damage, rendering repair and reconstruction rather slow.^14^-^16^ By inducing slight bone-bone interface motion, lumbar motion after surgery is bad for bone trabecula rebuilding and repair.^17^ Then slow repair aggravates the bone-bone interface motion, forming a vicious circle.^18^,^19^ Compared with small-sized bone graft granules, bulk graft bone granules have advantages in forming fiber joint and callus joint due to abundant bone trabecula structure with continuity and completeness. In this study, it is relatively difficult to measure the volume of the bones taken during the window opening process. In practice, we do not use the grinding and drilling window, but use the rongeur, bone knife, gun-shaped rongeur and Instruments such as nucleus pulposus perform vertebral fenestration, lateral crypt enlargement, and nerve root decompression. Bone the bones of different sizes with a rongeur, scissors, etc., and put together the bones of different diameters, stuff them into a 5ml disposable sterile plastic syringe, push the syringe plunger with the thumb or palm, and let the bones be exhausted. Squeeze gap becomes smaller. The method is rough, but it can conveniently and quickly estimate the amount of intervertebral bone graft, avoiding excessive bone grafting or insufficient bone grafting.

To sum up, different bone graft granule volumes hardly affected postoperative symptoms, but middle-sized bone graft granule (0.10cm^3^/granule) with postoperative imaging evaluation ability significantly augmented the postoperative intervertebral fusion rate and reduced the intervertebral space height loss. Thus, the findings pave the way for lumbar posterior pedicle screw internal fixation with Cage supporting bone graft fusion.

### Authors’ contributions

**SL** designed this study and prepared this manuscript.

**JH, CY & PT** collected and analyzed clinical data.
